# Are the Levels of Lipid Parameters Associated with Biometeorological Conditions?

**DOI:** 10.3390/ijerph16234636

**Published:** 2019-11-21

**Authors:** Rafał Skutecki, Iwona Cymes, Ewa Dragańska, Katarzyna Glińska-Lewczuk, Adam Buciński, Marek Drozdowski, Jerzy Romaszko

**Affiliations:** 1 School of Medicine, Family Medicine Unit, University of Warmia and Mazury in Olsztyn, 10-082 Olsztyn, Poland; rafal.skutecki@uwm.edu.pl; 2 Department of Water Resources, Climatology and Environmental Management, University of Warmia and Mazury in Olsztyn, 10-718 Olsztyn, Poland; iwona.cymes@uwm.edu.pl (I.C.); ewad@uwm.edu.pl (E.D.); kaga@uwm.edu.pl (K.G.-L.); 3 Department of Biopharmacy, Nicolaus Copernicus University in Toruń, Collegium Medicum in Bydgoszcz, 85-089 Bydgoszcz, Poland; kizbiofarmacji@cm.umk.pl; 4 Department of Laboratory Medicine, University of Warmia and Mazury in Olsztyn, 10-082 Olsztyn, Poland; m.drozdowski@szpital.olsztyn.pl

**Keywords:** lipids, cholesterol, thermal stress, cold stress, diet

## Abstract

Lipid disorders, especially hypercholesterolemia, are one of the most thoroughly investigated cardiovascular risk factors. Their correlation with biometeorological conditions has been reported, with authors stressing seasonal increases of total cholesterol (TC) levels, mostly occurring in winter. This study aims at determining the correlation between the level of lipid parameters (LP) and meteorological conditions, analyzing seasonal variations in LP levels, and attempting to answer the following questions: do changes in LP levels result from the organism’s response to cold or heat stress, or are they secondary to seasonal dietary variations? An observational study comprised ambulatory patients from the city of Olsztyn (Poland), for whom laboratory test were performed in 2016–2018, with 106,325 records of TC, high-density lipoprotein (HDL), and triglycerides (TG). LP levels were matched with atmospheric conditions on the day when the test was conducted and expressed by the universal thermal climate index (UTCI). We demonstrated seasonal increases of TC in cold stress (in wintertime) and of TG in heat stress (summer). The analysis of LP levels in specific periods revealed the increase of TC levels after holidays (i.e., Christmas and Easter) in men by 4.56%, and the increase of TG levels in women by 13.46% in the same period. Our results suggest the secondary, diet-dependent underlying cause of the observed changes. This work contributes to the discussion concerning the impact of biometeorological factors on LP levels and may be of significance when planning population-dedicated preventive activities.

## 1. Introduction

Lipid disorders, especially hypercholesterolemia, are one of the most thoroughly investigated cardiovascular risk factors. Their epidemiology, apart from genetic predispositions, depends on diet, national income, and many other factors [1,2]. Numerous studies and statistical analyses have been devoted to this subject.

Mean population lipid parameters (LP) values demonstrate seasonal variations. Higher mean total cholesterol (TC) values in wintertime were reported by the authors of the 2016 European Society of Cardiology (ESC) and European Atherosclerosis Society (EAS) guidelines for the management of dyslipidemias [3]. The authors of the guidelines based their conclusions on the results of the study conducted by Marti-Soler et al. [4], which indicated seasonal correlations of many cardiovascular risk factors, including higher TC values in wintertime in both hemispheres.

Vedel-Krogh et al. [5] reported seasonal changes in LP levels in the Danish population and demonstrated significantly higher TC and low-density lipoprotein (LDL) levels after Christmas. However, their data indicate that the observed change occurs not only in the post-Christmas period, but also in the entire winter season. This is also the conclusion reached by Nadif et al. [6], based on a very similar study devoted to seasonality in the French cohort. Seasonal variations in LP levels were also described by other authors. Ockene et al. [7] reported significantly higher TC and LDL levels in winter in females, and Janecki [8] demonstrated seasonal changes in LP values based on data collected in Poland, indicating that the shape of the variation curve for mean TC values may be different in different regions of the country and in different years. From our point of view, the most important correlation was revealed by Zhou et al. [9], who reported that the change of the average air temperature by 20 °C may result in a 20% change of the LP value. Zhou et al. noticed evident, temperature-dependent, seasonal variations of mean LP values, with the lowest TC and LDL levels observed in warm summer months. 

Despite these reports, it is still unclear if the increase of TC levels results from the organism’s response to cold stress, or if the observed change is correlative to seasonal dietary variations. Considering entire populations, in climatic zones characterized by evident seasonality, dietary habits associated with a given season of the year are related to a higher supply of calories in winter and a higher intake of fats, which may result in increases of LP levels [10,11]. For instance, Ersoy et al. [12] reported an increase in the consumption of high-energy food in winter months by 557 kcal daily in males and by 330 kcal in females (Turkey).

The question formulated earlier may be addressed either by conducting a planned prospective study or by changing the approach to the studied issue. If variations in LP levels depend on the exposure to cold/heat stress, they should also appear during short-term weather changes in a given season, especially since the dietary impact on LP levels is not immediate. Hence, the possible evidence for the correlation between a few days of exposure of the organism to cold/heat stress and LP levels might indirectly prove that the described link is direct rather than secondary and dependent on other factors [13]. In our view, such an approach may also minimize the impact of seasonal variations in physical activity on LP levels. In spring and summer, physical activity is generally higher by 15%–20% than in winter [14,15]. 

It should also be remembered that dietary habits are culture-dependent and may result in significant, periodical dietary modifications leading to changes in LP levels. This is especially true for different holidays related to a given country’s tradition. It is quite commonly known that, for instance, in Muslim culture during Ramadan (fasting), high-density lipoprotein (HDL) levels increase, and LDL levels decrease [16]. Periodical dietary modifications are also observed in Christian countries. A number of studies based on data from such countries reported weight gain in the Christmas period [17,18].

In Poland, Christmas celebrations begin on 24 December with Christmas Eve supper, traditionally consisting of 12 dishes, and last until 26 December. This is a time of family gatherings accompanied with hours of sitting at tables laden with food and eating. One Polish study, devoted to the calorie content of meals at Christmas time, reported an average intake of about 2400 kcal daily and an average weight gain by 420 g, the effect was comparable to that after Thanksgiving in the USA [19,20].

To describe local atmospheric conditions, a number of simple meteorological parameters may be used, such as: outside temperature (minimum, average, maximum), wind speed, atmospheric pressure, and many others. However, if we consider the level of energy required by a human organism, the biometeorological parameter of choice seems to be the universal thermal climate index (UTCI). This index comprehensively describes the impact of meteorological factors on the human organism and appears to be particularly useful for describing the organism’s response to thermal stress, including cold stress [21,22,23,24]. The UTCI derives from an analysis of human thermal balance, performed with the use of a multi-node model of human heat transfer [23]. The UTCI is a universal index due to its appropriateness for thermal assessments in all climates, seasons, and scales. Additionally, the UTCI is independent of personal characteristics, such as age, gender, specific activities, and clothing, and is more sensitive to changes in the following ambient stimuli: temperature, solar radiation, humidity, and wind speed [21,22]. It is calculated based on the following parameters: air temperature, water vapor pressure, relative humidity, wind speed, and expressed in degrees Celsius (°C) [25].

The aim of this work is to determine the correlation between LP levels and biometeorological conditions expressed with the UTCI, to analyze seasonal variations in LP levels, and to answer the following question: do changes in LP levels result from the organism’s response to cold/heat stress, or are they secondary to seasonal dietary variations?

## 2. Materials and Methods 

This observational study was based on laboratory and meteorological data collected in the period 2016–2018 in Olsztyn, Poland. Olsztyn has a population of about 180,000 inhabitants and is located in a cold climate-type zone without a dry season with warm summer (Dfb) [26]. In our climatic zone, December, January, and February are winter months, whilst June, July, and August are summer months. In Poland, ambulatory laboratory tests are free of charge when ordered by family doctors and specialists but may be also performed at the patient’s request, for payment. An average cost of the lipid profile test is low and amounts to about 20 PLN (5 EUR).

### 2.1. Study Population

An observational study comprised patients, aged between 18 and 99 years of age, for whom laboratory tests were performed (106,325 records in total). The results were obtained from two large laboratories located in Olsztyn: DIAGNOSTYKA limited liability company and the Municipal Hospital. The analysis involved the results of basic lipid tests: total cholesterol (TC) (n = 43,551), high-density lipoprotein (HDL) (n = 30,046), and triglycerides (TG) (n = 32,728). The analyzed results contain both the assays from prophylactic examinations and routine follow ups of patients, including those who take hypolipidemic drugs. For the sake of this study, no personal details concerning the patients were processed. Table 1 presents patient demographics.

### 2.2. Meteorological Data

To assess thermal stress, the UTCI values based on data recorded at 12.00 were used, calculated for consecutive days of 2016–2018. Input data were obtained from the Olsztyn weather station belonging to the Institute of Meteorology and Water Management. The obtained UTCI values were classified to appropriate thermal conditions classes, including cold stress class (UTCI ≤ 9.0 °C), thermoneutral conditions (UTCI from 9.1 to 26 °C), and heat stress class (UTCI > 26 °C) [25]. 

Cold stress dominated in the analyzed period, found for 52% (n = 570) of days, thermoneutral conditions occurred on 38.05% (n = 417) of days, and heat stress on 9.95% (n = 109) of days.

### 2.3. Special Situations

Consistent with the suggestion of Vedel-Krogh et al. [5], the impact of holidays (e.g., Christmas) on LP levels was analyzed. To this end, LP values 4 days before and 4 days after Easter, and analogously 4 days before and after Christmas, were compared. We assumed a 4 day comparison period because Easter is always on Sunday, followed by Easter Monday, a public holiday in Poland. A different (higher) calorie content of meals on off-work days was previously described, and could impact on the LP analysis [27]. 

To assess the impact of biometeorological conditions during the studied 3 years on the results of LP levels, we used 7 two-week long periods characterized by a significant change in meteorological parameters, in which the average UTCI value for the previous week was more than 10 °C lower, as compared to the following week, and 7 periods when the average UTCI value for the previous week was more than 10 °C higher than in the following week. We selected these periods to assess the impact of warmer and colder outdoor temperature on LP values in the patients.

### 2.4. Statistical Analysis

Statistical analysis was performed with the use of Statistica (data analysis software system) 13.3 version (TIBCO Software Inc., Palo Alto, USA). To assess the distribution of the analyzed variables, the Shapiro–Wilk test was used, and to assess the equality of variances, Levene’s test was employed. The comparison of weekly, monthly, and thermal variability (cold stress—thermoneutral conditions—heat stress) was performed with the analysis of variance (ANOVA) test and with the use of the Least Significant Difference (LSD) test as a post hoc procedure. When the equality of variance was not met, the Kruskal–Wallis test was used with multiple comparisons of median ranks. To compare the impact of holidays (Christmas, Easter) and colder/warmer outdoor temperature, Student’s *t*-test was used, and the Mann–Whitney U test was used when the equality of variances was not met. The statistical significance level was assumed at *p* ≤ 0.05.

## 3. Results

The mean TC level in our dataset was 203.18 mg% (standard deviation (SD) = 45.07), the mean value of TG amounted to 132.68 mg% (SD = 97.03), and of HDL, 61.08 mg% (SD = 17.96). The analysis of lipid data revealed a decrease of the mean TC level in consecutive years (Table 2).

The decrease of TC levels in consecutive years is not a correlative of the population undergoing laboratory tests ‘getting younger’ (Table 1). After limiting the study group to an age range of 40–60 years, statistical correlations remain the same as those presented in Table 2, and the age difference is not statistically significant. 

Mean TC and HDL values, in each analyzed year, were statistically significantly higher in females (*p* < 0.001), while the mean TG level was higher in males (Table 2). 

**Figure 1:**
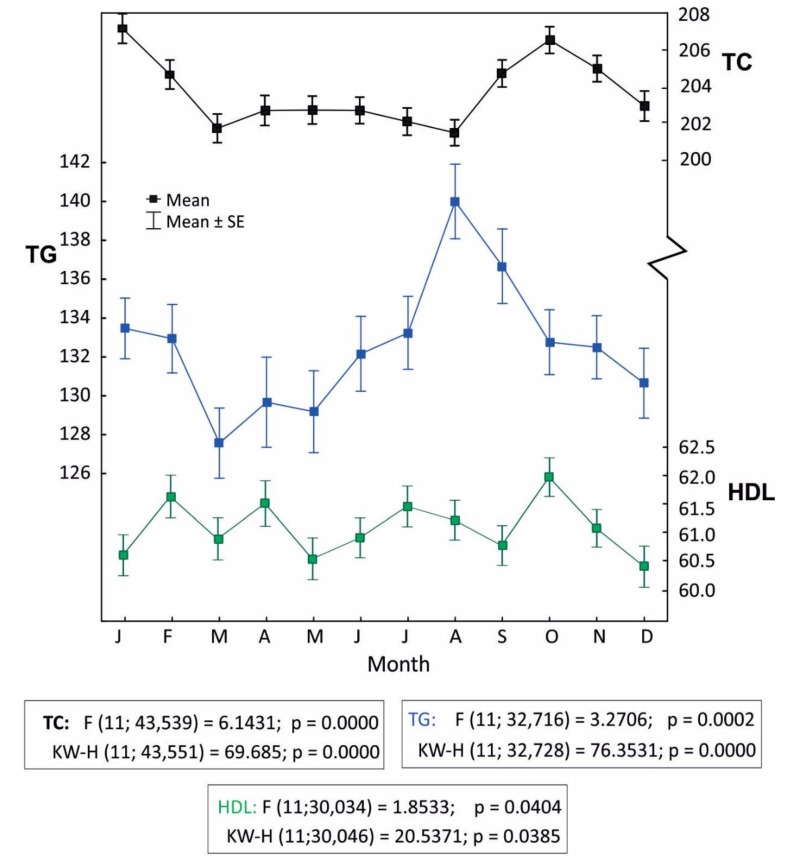
Monthly variations of lipid parameters (LP) levels. Data analysis was performed by one-way analysis of variance (ANOVA) (F-test) and Kruskall–Wallis (KW-H) test.

In the seasonal analysis, in a month-to-month assessment (Figure 1), some statistical correlations are noticeable, for instance: the highest mean TC levels in males occurred in October (203.79 mg%; SD = 46.04), and the lowest in May (197.02 mg%; SD = 45.70), the difference being statistically significant (*p* < 0.001). It should be remembered, however, that in the Polish climatic zone (Dfb), meteorological conditions in specific months are characterized by large variabilities in consecutive years. Seasonal variations of LP levels are presented in Figure 1. 

The decrease of TC levels in December and high TG values in August merit attention. In summer months (July–September), high TG values were observed more often (TG > 400 mg%), in total there were 150 cases (35%) and in August, 12% (n = 58).

The comparative analysis of LP levels in females and males under cold stress conditions, thermally neutral conditions, and heat stress, revealed statistically significant differences (Table 3). Patients in heat stress conditions had significantly lower (*p* < 0.001) mean TC values (TC = 200.49 mg/dL; SD 45.01) as compared to cold stress conditions (TC = 204.32 mg/dL; SD 45.43) 

The analysis of special situations—the impact of a short-term (one-week-long) change in weather conditions, performed for all 14 periods together, revealed higher TC values ‘on the cold side’ of the analysis. In seven periods when the weather became colder, TC increased substantially in females (204.95 mg% versus 208.67 mg%, *p* < 0.05), while in males, the difference was not statistically significant (200.04 mg% versus 202.1 mg% *p* = 0.34). 

A separate analysis of each special situation is presented in the supplementary file (S1), Table S1.

Based on the comparative analysis of mean LP levels before and after the holidays (Christmas and Easter), significantly higher mean TC values by 4.56% (*p* < 0.0023) and HDL by 7.25% (*p* < 0.0007) were observed in males in the post-holiday period, and significantly higher mean TG levels by 13.46% (*p* = 0.0210) in females in the post-holiday period, as compared to the levels before the holidays (Table 4). 

## 4. Discussion

A simple statistical analysis of two, or more, distant factors (in this study: LP levels and meteorological conditions) is always burdened with some risk of indirect correlations. For instance, a larger number of cardiovascular deaths in the winter season may be attributed to the direct response of the organism to cold stress (e.g., increase of arterial blood pressure), may be secondary to seasonal increases in respiratory tract infections, or may be associated with decreases in vitamin D3 levels in winter [28,29,30,31,32]. It may also be the result of any combination of these factors as well as many others. Higher TC levels in winter and lower in summer reported by Zhou et al., Vedel-Krogh et al., and Nadif et al. are generally consistent with our data [5,6,9]. The only, easily noticeable, difference is the decrease of TC levels in December. This decrease is contradictory to the hypothesis concerning the impact of cold stress on TC levels (Figure 1), especially since high values are revealed in January, and these are two consecutive months with similar meteorological characteristics. However, no contradiction occurs which regards dietary correlations. December precedes Christmas and is linked with culture-based limitations in food intake during advent time. In Poland, Catholics constitute about 90% of the entire population, yet only about 50% actively participate in religious events [33]. If the impact of religious factors here is probably insignificant, some culture-related habits may be of importance. January is the carnival month, when weddings and balls are organized, traditionally not taking place in December. 

The correlation presented in Table 3, that is higher TC levels in cold stress, is consistent with the reports quoted earlier, and is not contradictory to our hypothesis. Dietary habits in the population, historically related to the accessibility of specific foods and demands for energy, and thus calories, with an increase in food intake in autumn and winter, especially regarding fats and meat products, are well-known and well-investigated [11,34,35]. In our opinion, seasonal variations in dietary habits most likely contribute to variations in LP levels and are responsible for the correlations presented in Table 3. Hence, while not negating correlations, also evident in our dataset, between UTCI values and LP levels in annual, seasonal, and monthly calculations, we believe that the food correlation is one of the factors contributing to variations in LP levels. 

Yet, explanations referring to the direct response of the organism to cold/heat stress cannot be ignored either. It is commonly known that the organism’s responses to a drop in outdoor temperature involve increased basic metabolism, increased activity of the brown adipose tissue, and improved LP levels [36,37,38]. In the experiment conducted with a group of volunteers, described by van der Lans et al. [39], apart from the activation of the brown adipose tissue, an evident drop in TG levels was observed as a response to cold conditions. Based on a study involving a group of 19 vegetarians who remain on a stable diet independent of seasonal variations, Blüher et al. [40] draw far-reaching conclusions and suggest the presence of a seasonal rhythm of TC levels with increases in winter. However, our data, comprising population values of LP, indicate a significant responsibility of dietary factors with regard to this rhythm. The temporal association between the observed changes in LP levels and periods linked with a significant increase of food intake is difficult to overlook (Table 4). 

The analysis of special situations (Supplementary Table S1, Table 4), revealing an increase of TC levels after holidays in males and of TG in females, is consistent with dietary habits of the Polish population [41]. 

As for the ongoing discussion, conclusions that may be drawn from the response to a short-term change in weather conditions seem more important. It may be hypothesized that the increase of TC levels on ‘the cold side’ of the analysis in females (all intervals together) and the absence of this effect in males, may be related to a higher sensitivity of females to cold stress (the difference in the body surface to body mass ratio) [42]. This is similar, however, to the analysis presented in Table 3. A separate interpretation of each period with different thermal characteristics (Supplementary Table S1) does not justify any conclusions regarding a direct correlation between LP levels and atmospheric conditions. The response to warmer weather, presented on the right side of the table, can be practically ignored, and the response to colder weather (the left side of the table) is rather chaotic but consistent with a general tendency. 

High TG values in August are difficult to interpret (Figure 1). TG values are higher in heat stress conditions and thermoneutral conditions (Table 2), but only among males. Our dataset does not allow us to conclude why this is the case, however—consistent with our working hypothesis concerning the secondary, diet-dependent cause of biometeorological associations—this outcome may be linked with the summer holiday season in Poland and the associated increased intake of fatty foods (barbeques) and increased consumption of alcohol. This is, however, only a hypothesis, which we are unable to document. Despite our attempts, we were unable to obtain access to reports specifying monthly consumption of alcohol in Poland (most likely such reports do not exist). Our hypothesis is indirectly supported by the analysis of the number of cases with TG > 400 mg%. High TG levels are often found in patients with toxic alcohol-related liver damage [43]. In our dataset, such values most often occurred in summer months (July–September): 35% (n = 150) of cases, in August, 12% (n = 58).

Decreased mean TC levels in consecutive years (Table 2) may result from the changes in the treatment of patients with hyperlipidemia. New guidelines (presented in 2016) assuming significantly lower target LP levels both in primary and secondary prevention impacted on therapeutic practices [3]. Lower mean TC values in males as compared to females and higher mean TG in males as compared to females in population studies are quite characteristic for low-income and middle-income subregions compared to European countries [1]. The described differences are usually small. Gupta et al. [44] presented very similar correlations, simultaneously stressing that higher mean TC values in females appear after the age of 45 years. Since in our study the average age of females is statistically significantly higher than in males (e.g., in 2018, 55.71 versus 53.05 at *p* < 0.001), we consider these values as consistent with data from the available literature [1,45].

When planning this study, we primarily considered its practical dimension. Seasonal variations in LP levels lead to the question as to when, from the point of view of public health, primary preventive activities should be most beneficial, i.e., screening for hyperlipidemia. Because cold stress dominates (52%) in the Polish climatic zone, it appears that such activities should be undertaken in the autumn–winter season, excluding special situations (pre- and post-public holiday periods) which may influence the obtained population values. Unfortunately, the mentioned special situations are correlative to culture-dependent conditions and may prove different in other countries.

## 5. Conclusions

LP levels reveal seasonal variations. TC values are statistically significantly higher in cold stress conditions, i.e., in wintertime. However, these variations are most likely secondary to seasonal dietary changes. LP variations in the peri-holiday periods should be taken into consideration when planning screening examinations. 

## 6. Limitations

This study is based on a large dataset; however, the data come from one center (Olsztyn, Poland) collected over a relatively short time period (three years). This limitation was related to the change in laboratory assaying systems in both laboratories and changes in information technology systems that made it practically impossible to make use of earlier data. This study is, however, easily reproducible in a different center, most beneficially in a different climatic zone and culture. Its confirmation might end the discussion concerning the impact of weather conditions on LP values. The analysis does not consider LDL levels due to methodological reasons: some results are taken from the direct assay, the majority from Fridewald’s formula [46]. From a mathematical standpoint, in this approach, LDL is a processed value, secondary to input parameters. 

The factor that may interfere with the achieved results, yet lowering the values rather than inflating them, especially with regard to the analysis of cold stress, is the avoidance of thermal stress by remaining indoors. We are unable, however, to indicate the reason why this would occur in any specific winter month and not any other. Moreover, since the study is based on raw data referring to lipid parameters and on the material that did not contain any patients’ clinical details, such as: diet, drugs, co-existing diseases, etc., it is possible that its duplication conducted on a selected subpopulation may yield different results. Consequently, in our study, we operated only on the analysis of mean values. However, with such a large dataset collected in one region, our results may be considered to be population values.

## Figures and Tables

**Table 1:** Average age and gender of participants; assessment of the significance of differences between average values in subsequent years of the study.

Year	Total (SD; n)	Females (SD; n)	Males (SD; n)
2016	**56.84^ab^** (16.32; 26,177)	**58.27^ab^** (16.33; 15,352)	**54.80^ab^** (16.01; 10,825)
2017	**54.83^a^** (16.32; 39,645)	**55.79^a^** (16.53; 23,027)	**53.50^a^^c^** (15.92; 16,618)
2018	**54.65^b^** (16.37; 40,503)	**55.71^b^** (16.56; 24,382)	**53.05^b^^c^** (15.97; 16,121)

* values marked with the same letter in the superscript differ significantly statistically: analysis of variance (ANOVA) *p* < 0.05 (*p* < 0.001 in bold), comparisons in columns, SD: standard deviation.

**Table 2:** Mean values (±SD) of lipid parameters (mg%) in consecutive years.

Year	TC (mg%) (SD; n)	HDL (mg%) (SD; n)	TG (mg%) (SD; n)
**Total**			
2016	**208.64^ab^** (45.37; 10,986)	60.77^a^ (18.11; 7256)	131.09^a^ (91.00; 7935)
2017	**203.52^ac^** (44.71; 16,262)	**60.75^b^** (17.79; 11,176)	131.55^b^ (93.62; 12,207)
2018	**199.16^bc^** (44.82; 16,303)	**61.60^ab^** (18.02; 11,614)	134.77^ab^ (103.71; 12,586)
**Males**			
2016	**205.37^de^** (44.22; 4682)	52.85 (15.09; 2924)	147.69^c^ (111.47; 3219)
2017	**200.18^df^** (45.16; 6858)	52.91 (14.99; 4665)	149.79 (121.82; 5095)
2018	**194.74^ef^** (45.37; 6429)	53.24 (15.00; 4634)	154.60^c^ (139.24; 5058)
**Females**			
2016	**211.06^gh^** (45.14; 6304)	66.12^c^ (18.03; 4332)	119.76 (71.67; 4716)
2017	**205.96^gi^** (44.22; 9404)	66.36^d^ (17.52; 6511)	118.49^d^ (63.29; 7112)
2018	**202.04^hi^** (44.23; 9874)	67.16^cd^ (17.70; 6980)	121.45^d^ (67.20; 7528)

* values marked with the same letter in the superscript differ significantly statistically-ANOVA p < 0.05 (p < 0.001 in bold), comparisons in columns (in subgroups by gender). Total cholesterol (TC), high-density lipoprotein (HDL), and triglycerides (TG). SD: standard deviation.

**Table 3:** Mean values (SD) and number of lipid parameters (mg%) and the universal thermal climate index (UTCI) depending on thermal stress.

	Cold Stress (SD; n)	Thermoneutral Conditions (SD; n)	Heat Stress (SD; n)
**Total**			
TC	**204.32^ab^** (45.43; 21,803)	202.47^ac^ (44.58; 17,020)	**200.49^bc^** (45.01; 4728)
HDL	60.89 (27.85; 15,340)	61.14 (18.18; 11,468)	61.82 (17.69; 3238)
TG	131.92^a^ (90.14; 16,730)	132.36^b^ (100.02; 12,468)	137.42^ab^ (115.88; 3530)
**Females**			
TC	**206.73^ab^** (45.29; 12,593)	205.20^ac^ (43.89; 10,058)	**203.02^b^^c^** (43.80; 2931)
HDL	66.55 (17.76; 8896)	66.49 (17.81; 6930)	67.36 (17.17; 1997)
TG	119.73 (67.72; 9707)	120.26 (67.72; 7474)	119.87 (66.21; 2175)
**Males**			
TC	**201.01^ab^** (45.42; 9210)	**198.53^a^** (45.27; 6962)	**196.37^b^** (46.64; 1797)
HDL	53.08 (14.75; 6444)	52.97 (15.51; 4538)	52.91 (14.60; 1241)
TG	**148.77^a^** (111.93; 7023)	**150.46^b^** (133.75; 4994)	**165.59^a^^b^** (163.30; 1355)

* values marked with the same letter in the superscript differ significantly statistically: ANOVA *p* < 0.05 (*p* < 0.001 in bold), comparisons in lines.

**Table 4:** Changes (%) in the mean value (±SD) of TC, TG, and HDL levels (mg%) for males and females in 4-day periods before and after holidays.

	Before Holidays	After Holidays	Change (%)	*p*
**Males** (SD; n)				
TC	198.55 (43.74; 461)	207.6 (46.49; 473)	4.56	<0.0023
HDL	50.49 (14.02; 323)	54.15 (14.27; 388)	7.25	<0.0007
TG	156.82 (176.56; 353)	158.58 (105.25; 418)	1.12	<0.8644
**Females** (SD; n)				
TC	203.18 (47.19; 504)	205.18 (41.58; 625)	0.98	<0.1803
HDL	67.75 (18.29; 358)	66.6 (17.13; 482)	−1.70	<0.3519
TG	111.11 (73.55; 390)	126.07 (110.80; 523)	13.46	<0.0210

## Supplementary Materials

The following are available online at https://www.mdpi.com/1660-4601/16/23/4636/s1, Table S1: Effect of UTCI change (hot/cold and cold/hot) between consecutive weeks and holidays (Christmas and Easter periods) on lipid parameters (LP).
