# Supplementary material for: Are the Levels of Lipid Parameters Associated with Biometeorological Conditions?
Source: Int J Environ Res Public Health. 2019 Nov 21;16(23):4636. doi: 10.3390/ijerph16234636 (PMC6926572; doi:10.3390/ijerph16234636)
Supplement: Supplementary file 1 [file ijerph-16-04636-s001.pdf]

Table S1. Effect of UTCI change (hot/cold and cold/hot) between consecutive weeks and holidays (Christmas and Easter periods) on lipid parameters (LP).

| | hot/cold | | | | | | | cold/hot | | | | | | | before/after holidays |
|---|---|---|---|---|---|---|---|---|---|---|---|---|---|---|---|
| period | 10-16.09.2016<br>17-23.09.2016 | 27.09-3.10.2016<br>4-10.10.2016 | 30.03-.04.2017<br>6-12.04.2017 | 17-23.10.2017<br>24-30.10.2017 | 14-20.06.2018<br>21-27.06.2018 | 17-23.09.2018<br>24-30.09.2018 | 15-21.10.2018<br>22-28.10.2018 | 15-21.06.2016<br>22-28.06.2016 | 8-14.02.2017<br>15-21.02.2017 | 19-25.03.2017<br>26.03-<br>1.04.2017 | 6-12.05.2017<br>13-19.05.2017 | 28.03-.04.2018<br>4-10.04.2018 | 27.06-.07.2018<br>4-10.07.2018 | 23-29.10.2018<br>30.10-<br>5.11.2018 | |
| both sexes | | | | | | | | | | | | | | | |
| TC | i | i | ns | ns | i | ns | ns | ns | ns | ns | ns | ns | ns | ns | i |
| TG | ns | ns | ns | ns | ns | ns | ns | ns | ns | ns | d | ns | ns | ns | ns |
| HDL | ns | ns | ns | ns | ns | ns | ns | ns | ns | ns | ns | ns | ns | ns | i |
| males | | | | | | | | | | | | | | | |
| TC | ns | i | ns | ns | ns | ns | ns | ns | ns | ns | ns | ns | ns | ns | i |
| TG | ns | ns | ns | ns | ns | ns | ns | ns | ns | ns | ns | ns | ns | ns | ns |
| HDL | ns | ns | ns | ns | ns | ns | ns | ns | ns | ns | ns | ns | ns | ns | i |
| females | | | | | | | | | | | | | | | |
| TC | i | ns | ns | ns | i | ns | ns | ns | ns | ns | ns | ns | ns | ns | ns |
| TG | ns | ns | ns | ns | ns | ns | ns | ns | ns | ns | ns | ns | ns | ns | i |
| HDL | ns | ns | ns | ns | ns | ns | ns | ns | ns | ns | ns | ns | ns | ns | ns |

i-increase, d-decrease, n.s. – not significant p< 0,05
